# Chronic kidney disease as a cardiovascular risk factor: lessons from kidney donors

**DOI:** 10.1016/j.jash.2018.04.010

**Published:** 2018-07

**Authors:** Anna M. Price, Nicola C. Edwards, Manvir K. Hayer, William E. Moody, Richard P. Steeds, Charles J. Ferro, Jonathan N. Townend

**Affiliations:** Birmingham Cardio-Renal Group (University of Birmingham, Institute of Cardiovascular Sciences), Queen Elizabeth Hospital, Edgbaston, Birmingham, United Kingdom

**Keywords:** Cardiac, living kidney donors, transplant, mortality

## Abstract

Chronic kidney disease (CKD) is a major risk factor for cardiovascular disease but is often associated with other risks such as diabetes and hypertension and can be both a cause and an effect of cardiovascular disease. Although epidemiologic data of an independent association of reduced glomerular filtration rate with cardiovascular risk are strong, causative mechanisms are unclear.

Living kidney donors provide a useful model for assessing the “pure” effects of reduced kidney function on the cardiovascular system. After nephrectomy, the glomerular filtration rate ultimately falls by about one-third so many can be classified as having chronic kidney disease stages 2 or 3. This prompts concern based on the data showing an elevated cardiovascular risk with these stages of chronic kidney disease. However, initial data suggested no increase in adverse cardiovascular effects compared with control populations. Recent reports have shown a possible late increase in cardiovascular event rates and an early increase in left ventricular mass and markers of risk such as urate and albuminuria. The long-term significance of these small changes is unknown. More detailed and long-term research is needed to determine the natural history of these changes and their clinical significance.

## Highlights

• Subjects with chronic kidney disease (CKD) have an elevated risk of cardiovascular disease.
• Kidney donors have a reduced glomerular filtration rate and biochemical changes similar to CKD subjects.
• Most studies of donors have not shown an elevated risk of death or cardiac disease.
• Donors have structural and functional cardiovascular changes similar to early CKD.
• The clinical significance of these changes is currently unknown.

## Introduction

In the United Kingdom, almost half of kidney transplants are now from living kidney donors.^1^ The inevitable reduction in kidney function after uninephrectomy raises the possibility of adverse cardiovascular effects given the graded association of estimated glomerular filtration rate (eGFR) and cardiovascular risk, which appears to begin at an eGFR of 60–75 mL/min/1.73 m^2^.^2^ At 5 years after donation, up to a third of patients can be expected to have an eGFR of less than 60 mL/min/1.73 m^2^ using the modification of diet in renal disease or Chronic Kidney Disease Epidemiology Collaboration equations.^3^

Detailed studies of donors show small but significant structural and functional changes in the cardiovascular system at 1 year after nephrectomy.^4^, ^5^ In addition, a single but carefully designed study appears to show a late rise in adverse cardiovascular events.^6^ Studies of living kidney donors appear to be a good approach to disentangling the complex association of renal and cardiovascular disease allowing important pathophysiological information on the mechanisms of the association of chronic kidney disease (CKD) and cardiovascular disease to be gained.

## Mortality and Cardiovascular Events

Findings from multiple studies with up to 40 years of follow-up have shown no evidence of reduced survival compared with the general population, and some have reported better life expectancy (please see Supplementary Table S1 online).^7^, ^8^, ^9^, ^10^, ^11^ Most are single-center reports and describe health event rates far lower than the general population, although, importantly, the control data were often derived from populations containing large numbers of subjects who would not have been fit to donate.^9^ In an attempt to overcome this, Garg *et al.*^12^ used a matched cohort study to compare donor death and cardiovascular event rates with the “healthiest general population” and excluded those with conditions that would have precluded donation. Reassuringly, the combined end point of death and adverse cardiovascular events was lower in donors than controls, and the risk of cardiovascular events alone was not significantly different.^12^ Further support comes from a large study using US registry data comparing survival in over 80,000 donors with that of a matched cohort of 9364 participants without CKD drawn from the third National Health and Nutrition Examination Survey (NHANES).^7^ Over a median follow-up of 6.3 years, mortality among donors was not different to controls stratified by age, sex, and race.^7^

There are a number of limitations of these studies. First, the short durations of follow-up means that increased long-term cardiovascular risk cannot be excluded. To date, most studies have median follow-up periods of 6–8 years.^7^, ^12^, ^13^ Second, the influence of race on cardiovascular outcomes after kidney donation is unclear. Most of the outcome data are based on predominantly Caucasian populations such as those in Canada and Norway.^6^, ^12^ There is a need for mortality studies on black, Hispanic, and Asian patients, especially given the increased risk of hypertension in these groups.

Concerns relating to possible long-term adverse effects of donation arose in 2014 in an article examining 15-year outcomes in 1901 Norwegian donors and 32,621 control patients who were potentially eligible for donation.^6^ The hazard ratios for all cause death (1.30 [95% confidential intervals {CI} 1.11–1.52]), cardiovascular death (1.40 [95% CI 1.03–1.91]), and end-stage renal disease (ESRD) (11.38 [95% CI 4.37–29.63]) were significantly increased in donors with curves diverging after about 10 years.^6^ Limitations of this study include exclusion of marginal donors with comorbidity such as obesity, an older donor group (8 years) than controls, and longer follow-up of donors compared with controls.^6^, ^14^ In addition, the rural area of Norway used to conduct the study has an unusually high life expectancy, and most living kidney donors (including all who developed ESRD with its attendant high cardiovascular risk) were genetically related to the recipient.^14^ Nevertheless, these data are at least cause for concern and should give rise to more intensive long-term follow-up of donor populations around the world. It is impossible to exclude with certainty that a reduction in glomerular filtration rate (GFR) of any cause, including donation, may lead to an increase in adverse cardiovascular events.

A major problem with studies using non-mortality end points in living kidney donors is surveillance bias.^13^ Higher rates of hypertension and proteinuria in donors may be a result of more intensive medical review.^13^ Reese *et al.*^13^ found that donors made more visits to primary care and had more diagnosed non-melanoma skin lesions; both findings are suggestive of this form of bias. This reinforces the need for well-controlled prospective studies of adequate duration.

## Vascular Changes

### Hypertension

Most patients with CKD are hypertensive but it is not clear if this is a universal finding when GFR is reduced. There has been suspicion for many years that donors have excess rates of hypertension and albuminuria but the quality of evidence is poor and reports are inconsistent.^15^ A meta-analysis of 48 studies found that it was not possible to assess the risk of hypertension requiring treatment as none of the primary studies had an adequate sample size to detect a 1.5-fold increase in risk after donation with at least 80% statistical power.^16^ Thus, change in blood pressure (mm Hg) is frequently used as an intermediary marker for increased risk of hypertension.^16^ Of the 10 studies that had a control group and a follow-up of over 5 years, there was an increase in blood pressure of about 6 mm Hg systolic and 4 mm Hg diastolic when compared with healthy adults with similar age, sex, and ethnicity.^16^ Garg *et al.* also found that donors were more likely to be diagnosed with hypertension (defined using diagnostic codes on outpatient or discharge paperwork) than controls (16.3% vs. 11.9%, hazard ratio 1.4); however, there is a strong possibility of surveillance bias.^9^

There are many flaws in these studies; most were retrospective and few used contemporaneous control groups that were followed up in a similar way to donors.^15^ The transplant community can be criticized for a lack of quality prospective long-term studies of blood pressure in living kidney donors but there are significant obstacles. Not only are such studies expensive and difficult to perform, particularly with respect to finding appropriate controls, but live donor transplants are often carried out in large hospital centers involving long traveling times.^6^, ^15^ In Korea, for example, just 11% of patients were followed up despite over 80% of kidney transplantation in that country involving live donors.^17^

Data from 24-hour ambulatory blood pressure studies are mixed. In a prospective controlled observational study, Kasiske *et al.*^18^ found no statistical difference in ambulatory blood pressure values or in night-time “dipping” at 36 months between 135 well-matched controls and 126 donors. By contrast, data from 1214 donors in the mandatory Swiss lifelong donor follow-up has raised concern.^15^ Among initially normotensive donors, 43.1% developed hypertension diagnosed by ambulatory blood pressure monitoring within the 10-year follow-up period.^15^ Hypertension was defined as a systolic of greater than 140mm Hg and/or a diastolic of greater than 90 mm Hg or the use of an antihypertensive medication.^15^ There was no control group, so conclusions are difficult to draw but using the Framingham hypertension risk score, it was estimated that by 12 months, nephrectomy had increased the risk of hypertension by 3.64 times.^15^ The influence of race on rates of hypertension and other morbidities requires much more investigation. To date, the best data comes from a retrospective US study of 4650 living kidney donors.^19^ Postnephrectomy events were compared with NHANES data from the general population with a median follow-up of 7.7 years.^19^ Thirteen percent of the group were black and 8% Hispanic.^19^ The overall prevalence of hypertension at 5 years was 17.8% but this was increased by 52% for blacks and 36% for Hispanics compared with white donors, exceeding what would be expected in the general population in both Hispanic and black patients over the age of 55 years.^19^ The definition of hypertension was based on billing claims, pharmacy claims, and antihypertensive drug category codes.^19^

In a number of studies, blood pressure variability rather than blood pressure alone has been linked to cardiovascular mortality and progression of renal disease.^20^, ^21^ Ternes *et al*.^22^ studied 193 donors and 196 controls as part of the prospective Assessing Long-Term Outcomes in Living Kidney Donors study. There was no difference in blood pressure coefficient of variance 12 month after nephrectomy compared with controls.^22^ In summary, despite years of study, it is still not possible to draw safe conclusions on whether the reduction in GFR caused by kidney donation causes an increase in blood pressure. This may be because there is no renal cortical damage or ischemia in kidney donors; the circulating renin-angiotensin system is probably not activated.^4^, ^5^ This lack of association between living kidney donors and increased risk of HTN benefits studies investigating the influence of a reduced GFR on the cardiovascular system as it eliminates the possible confounding effects of high blood pressure. The caveat, however, is that if blood pressure is a major distinguishing feature between donors and patients with chronic kidney disease, findings in kidney donors may not apply to those with CKD.

### Pre-eclampsia and Gestational Hypertension

Patients with CKD are at higher risk of developing pre-eclampsia during pregnancy and at an increased severity compared with controls.^23^ This is of importance with respect to long-term cardiovascular health as pre-eclampsia confers a 12-fold increased future risk of cardiovascular disease.^24^ Studies investigating risk of pre-eclampsia in living kidney donors are mainly retrospective, observational, and reliant on patient self-reporting. Ibrahim *et al.*^25^ reported on 1085 living kidney donors with 3213 pregnancies. Pregnancies after donation were associated with a lower rate of full-term deliveries (73.7% vs. 84.6%).^25^ Donors also had higher rates of gestational hypertension (5.7% vs. 0.6%) and pre-eclampsia (5.5% vs. 0.8%) after donation than before donation.^25^ Gestational hypertension was defined as a need for treatment during pregnancy only (not before or after).^25^ Maternal, fetal, and pregnancy outcomes were, however, similar to the general population, and the influence of patient bias recall cannot be discounted.^25^ In a similar study, Reisaeter *et al.*^26^ also used questionnaires to review over 100 living kidney donors and found higher pre-eclampsia rates after donation than before (5.7 vs. 2.6%), although maternal age, a major confounder, could not be entirely accounted for in multivariable modeling due to the low event rate. As the pregnancy complications were recorded by clinicians, this data may be more accurate.^26^ In a retrospective cohort study of 85 female living kidney donors and 131 pregnancies, Garg *et al.*^27^ matched donors with controls in a 1:6 ratio for number of pregnancies, time to pregnancy, age, income, and urban/rural background. Gestational hypertension and pre-eclampsia (defined by diagnostic codes after clinical assessment) were more than twice as common in living kidney donors than controls.^27^ In a systematic review by the Kidney Disease Improving Global Outcomes work group, Slinin *et al.*^28^ concluded that women of child-bearing age should be informed of an increased risk as part of the consent process. On current evidence, it appears that kidney donation, like CKD, increases the risk of pre-eclampsia.

### Arterial Stiffness

Pulse wave velocity (PWV) is the gold standard non-invasive measure of aortic stiffness.^29^ It is elevated in CKD and a strong predictor of cardiovascular risk in CKD and a variety of other diseases.^30^ There are several studies of the effects of kidney donation on arterial stiffness but many are small uncontrolled pilot studies from which safe conclusions cannot be drawn. Fesler *et al.*^29^ showed no change in PWV or any other marker of arterial stiffness in a study of 45 donors before and 1 year after donation without a control group. By contrast, a cross-sectional study of 101 Lebanese kidney donors demonstrated that PWV was 10% higher than healthy controls with a similar age and sex distribution (although not screened to be “donor eligible”).^31^

It is estimated that the required sample size to adequately power a study to determine a 0.4 m/s change in PWV is over 350 patients per group.^30^ Because there are no studies of this size, it is unsurprising that the literature is inconsistent. In 2012, the Effect of A Reduction in glomerular filtration rate after NEphrectomy on arterial STiffness and central hemodynamics study began that has a prospective, multicenter, controlled longitudinal design.^30^ There is an ambitious aim of recruiting 400 donors and controls, which would allow sufficient statistical power to detect very small changes of the order of 0.2 m/s.^30^ The results are expected in 2018.^30^

An alternative method of measuring arterial stiffness is to use aortic distensibility, the change in cross-sectional area (usually measured by cardiac magnetic resonance [CMR]) per unit change in pressure. This has been used in a number of studies and is of prognostic value.^4^ In a prospective controlled study, distensibility was reduced in donors compared with controls at 12 months from nephrectomy.^4^ Reduced aortic distensibility has also been seen in patients with early-stage CKD.^32^

### Cardiac Structure and Function

Several studies have investigated whether human kidney donation causes structural and functional change in the left ventricle.^4^, ^5^, ^33^ Moody *et al.*^4^ studied 68 donors and 56 equally healthy controls (many of whom were worked up for donation but did not donate). At 12 months, there was an increase in left ventricular (LV) mass measured by CMR in donors but not controls.^4^ Global circumferential strain was also decreased indicating early changes in systolic dysfunction.^4^ There was no change in blood pressure measured by ambulatory monitoring and no association between change in LV mass and changes in blood pressure.^4^ In a similar but uncontrolled and smaller study also using CMR, Altmann *et al.*^5^ studied 23 living kidney donors and found that LV mass had increased at 12 months without change in office blood pressure. In a small cross-sectional echocardiographic and CMR study, 15 Italian donors were compared with age- and sex-matched healthy controls from the United States at a median of 8.4 years (minimum of 5 years) from donation.^33^ Most measures of LV geometry and function were not different in donors and controls but donors did exhibit abnormalities of LV apical rotation and torsion.^33^ By contrast, Hewing *et al.* also studied 30 living kidney donors at baseline and 12 months after donation using 2D speckle tracking echocardiography and found no significant differences in left or right ventricular function.^34^

In summary, there are few studies investigating cardiac structural and functional change after kidney donation. The studies that do exist have small sample sizes. Current evidence indicates that kidney donation results in small changes in cardiac structure and function. Whether these changes are sustained and are associated with an increase in cardiovascular risk is not known. Well-controlled follow-up studies with serial cardiac investigations are required.^35^

### Biochemical Changes

Traditional well-established risk factors for cardiac disease have been investigated in living kidney donors including the propensity to develop glucose intolerance, lipids, and the level of proteinuria compared with controls.

### Lipids and Glucose Tolerance

In a prospective study of 182 donors compared with 173 controls (also suitable for donation), there was no significant difference in lipid profiles including high-density cholesterol, low-density cholesterol, triglycerides, or lipoprotein(a) at 3 years.^18^ The subjects also underwent both a Haemoglobin A1c and “the homeostasis model assessment of insulin resistance” (HOMA-IR).^18^ Although both increased over time, there was no difference between the donors and controls.^18^

### Proteinuria

Proteinuria is an independent risk factor for cardiovascular mortality in the general population and patients with CKD.^2^ Recent studies have also demonstrated an increased prevalence of microalbuminuria.^4^, ^15^ Thiel *et al.*^15^ for example found that albumin to creatinine ratio (ACR) increased from 1.2 ± 2.7 to 1.9 ± 10.7 mg albumin/mmol creatinine in donors, and the prevalence of microalbuminuria increased from 4.8% to 10.4% over 10 years with a strong association with the development of hypertension. Moody *et al* also found that donors had a significantly raised prevalence of microalbuminuria compared with healthy controls at 12 months (odds ratio, 3.8 [95% CI, 1.1–12.8]; *P* = .04).^4^ This effect may be progressive; in a 3-year prospective study of living kidney donors and matched controls, Kasiske found a gradual rise in ACR in donors, which did not occur in controls.^18^

### Renin-Angiotensin-Aldosterone System

The importance of this system in CKD is emphasized by the efficacy of aldosterone-converting enzyme (ACE inhibitor) and angiotensin receptor blocker drugs in the control of hypertension and reduction in proteinuria and disease progression.^36^ Although this is thought to be an important mechanism of cardiovascular and renal damage in CKD, it may be one of many pathological pathways. Living kidney donors show no evidence of elevated concentrations of circulating renin or aldosterone and yet have evidence of cardiovascular damage including increased LV mass and reduced aortic distensibility.^4^, ^33^

Although circulating levels of renin and aldosterone have not been identified, there is some evidence of intrinsic activation.^37^ Kendi *et al*. used a novel method of investigating activation of the renin-angiotensin-aldosterone system in living kidney donors by studying urinary angiotensinogen before and after donation.^37^ Urinary angiotensinogen is considered a marker of intrarenal renin-angiotensin-aldosterone system activation and was five times higher at 12 months after donation compared with baseline.^37^ The study however only included 20 patients, and there was no control group.^37^

### Metabolic Bone Abnormalities

In a prospective controlled study, biochemical changes were examined in 201 donors and 198 controls at 6 months after donation.^38^ There was a large (23%) increase in parathyroid hormone (PTH) in this cohort; this increase was confirmed by Moody *et al*. in their prospective study of donors at 12 months.^4^ Parathyroid hormone may be an important mediator of left ventricular hypertrophy (LVH). It has been shown to be independently related to LVH in patients after aortic valve replacement, in patients with ESRD on hemodialysis and in the general population.^39^, ^40^, ^41^, ^42^

Fibroblast growth factor 23 (FGF23) also has an important role in bone metabolism and rises significantly in CKD.^43^ Concentrations of FGF23 are associated with increased LV mass in patients with CKD and animal and cellular work suggests a powerful hypertrophic effect on the myocardium.^44^ Expression of FGF23 receptors increase in the hearts of those with CKD and it is associated with LVH.^45^ FGF23 has been found to increase both after nephrectomy and compared with controls in a number of donor studies^4^, ^46^, ^47^, ^48^ although there are some inconsistencies which may be related to the use of different assays.^43^, ^49^

Klotho is a transmembrane protein associated with FGF23 signaling.^50^ Soluble klotho is cleaved and released into the circulation or urine.^50^ A reduction in α-klotho occurs in early CKD and is associated with accelerated aging.^43^, ^49^ A reduction in klotho is associated with cardiac remodeling and fibrosis.^51^ There have been two small studies investigating the effect of kidney donation on α-klotho with divergent results. Ponte *et al.* found an acute reduction in circulating klotho levels after serial measurements at 0, 1, 2, and 3 days after donation in 27 living kidney donors.^49^ Klotho levels remained lower than baseline at both 180 and 360 days after donation but had risen since the immediate postoperative period.^49^ In contrast to a cross-sectional study of 35 subjects at 5 years after donation, Thorsen *et al*.^43^, ^49^ found no difference compared with healthy controls. Taken together, these studies suggest that klotho levels may decline acutely after donation recovering to baseline in the long term but further studies are needed to draw firm conclusions.^43^

### Uric Acid

Uric acid is a result of purine metabolism and largely exists as urate.^52^ Although it has a powerful role as an antioxidant within serum, it has the potential to become an intracellular pro-oxidant agent.^52^ It has been shown to impair endothelial nitric oxide production and to cause inflammation and proliferation in smooth muscle by the NF kappa B pathway.^53^, ^54^ Over 70% of uric acid is excreted by the kidney and serum concentrations are therefore almost invariably raised in patients with CKD.^55^ In large population studies, elevated uric acid is associated with both hypertension and adverse cardiovascular outcomes.^56^, ^57^, ^58^, ^59^ It is therefore unsurprising that there is increasing interest in its role as a possible causative agent in the development of cardiovascular disease in patients with CKD. Although cause and effect has been difficult to establish, the importance of the role of uric acid is that it is a potentially modifiable risk factor.^60^, ^61^ Kao *et al.*^60^ showed that allopurinol reduced LV mass and improved both endothelial dysfunction and arterial stiffness in patients with early-stage CKD. Long-term use of allopurinol also improved both endothelial function and eGFR in patients with CKD.^62^ A recent meta-analysis of 16 trials concluded that uric acid–lowering therapy has a positive effect on both kidney function and also reduced cardiovascular events.^61^

In kidney donors at 1, 2, and 3 years, serum uric acid was elevated compared with controls meeting criteria for donation.^4^, ^18^ In a small prospective cohort study of 20 living kidney donors, uric acid levels decreased immediately after nephrectomy only to subsequently rise and remain high throughout the 12-month study.^37^ Over the long term, donors are more likely than controls to be newly diagnosed with gout and to be commenced on treatment with allopurinol or colchicine.^63^ In a small study of 42 living kidney donors, uric acid correlated with indoxyl sulfate and p-cresyl sulfate.^64^ These uremic toxins have potential importance as they have been found to be associated with increases in carotid intimal thickness and markers of endothelial dysfunction in donors.^64^

### Novel Cardiovascular Biomarkers

A variety of other biomarkers of cardiac disease have been found to be deranged in CKD and associated with cardiac events, death, and renal progression.^46^, ^65^ The data examining these biomarkers in donors are summarized online (please see Supplementary Table S2 online).

## Conclusion

**Figure 1:**
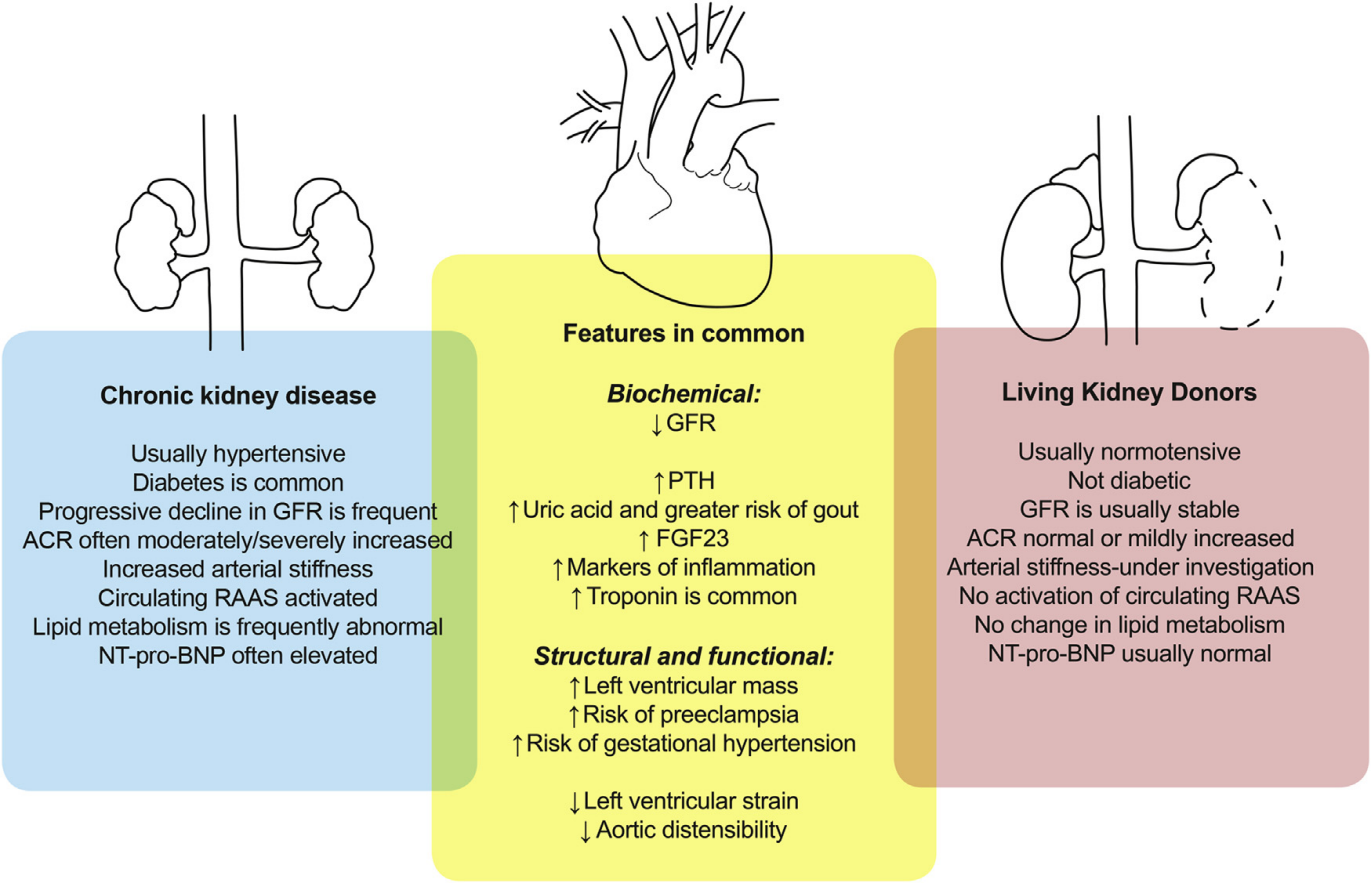
A comparison of donors and patients with CKD. Features in common give us valuable mechanistic information for possible mediators of cardiac disease.^3^, ^4^, ^5^, ^8^, ^15^, ^18^, ^25^, ^26^, ^27^, ^30^, ^33^, ^38^, ^46^, ^47^, ^48^, ^63^, ^65^, ^66^ ACR, urine albumin to creatinine ratio; FGF23, fibroblast growth factor 23; GFR, glomerular filtration rate; PTH, parathyroid hormone; RAAS, renin-angiotensin-aldosterone system; NT-proBNP, N terminal pro-brain natriuretic peptide.

Although there is evidence of an increase in long-term cardiovascular risk in living kidney donors from a single article,^6^ other studies have found no such effect, and further high-quality work is urgently required. Reassuringly, if the risk is increased, the level of this increase in risk is small with absolute risks remaining much lower than those of the general population.^12^ Effects on blood pressure and risk of hypertension remain uncertain but there is evidence from more than one study of changes in cardiac and vascular structure and function. As there was no change in blood pressure in these studies it appears likely that circulating factors associated with a decline in kidney function cause hypertrophic effects on the myocardium. Possibilities include uric acid, PTH, and FGF23, but the changes after donation are complex, and there may be other influences. Consequently, kidney donors have already provided us with valuable insights into the pathophysiology of cardiorenal disease by allowing examination of the isolated effects of a reduction in GFR (see Figure 1 for a diagrammatic summary).

These intriguing data have prompted several groups worldwide to enroll kidney donors in further prospective studies. The possibility of investigating causal mechanisms by using specific pharmacologic interventions in willing volunteer donor subjects arises. This might provide valuable mechanistic information on mediators of cardiac disease in those with CKD.

## Figures and Tables

**Table S1:** Summary of mortality studies in kidney donors

Reference	Date	Patient Numbers	Control Group	Matched Controls?	Follow up	Ethnicity/Age of Donors	Single Center?	Outcome	Mortality in Donors∗
Garg.^9^	2008	Donors = 1278 Controls = 6359	Health administration data	Yes—age, sex, income, and number of physician visits.	Mean 6.2 y.	92% Caucasian 5% Asian <3% black Canada	No	No differences in either mortality or cardiovascular disease events (1.3% vs. 1.7%; hazard ratio 0.7)	–
Ibrahim.^8^	2009	Donors = 3698	Life table data	Yes—age, sex, and race. 255 matched 1:1	40 y.	United States	Yes	Survival was similar to controls	–
Segev.^7^	2010	Donors = 80,347 Controls = 9364	Third cohort of National Health and Nutrition Examination Survey (NHANES III)	Yes—1:1 based on comorbidities.	Median 6.3 y.	13.1% black 12.3% Hispanic United States	No	Mortality among donors was no higher than controls even when stratified by age, sex, and race	–
Mjoen.^10^	2012	Donors = 2269 Controls = 6807	General population statistics	Yes—Age, gender, and year of birth. 3:1 match	Median 14.3 y.	Mean age 47 y 41% male Norway	Yes	Both overall and cardiovascular mortality was lower for donors	˅
Garg.^12^	2012	Donors = 2028 Controls = 20,280	“Healthiest general population” Excluded those conditions that precluded donation.	Yes—age, sex, income, and residence 10:1 match	Median 6.5 y.	Median age 43 y. Likely Caucasian, Ontario, Canada	Yes	Risk of death or major cardiovascular events was lower in donors with a hazard ratio of 0.66.	˅
Reese.^13^	2014	Donors = 3368 Controls = 3368	Healthy older patients in the Health and Retirement Study	Yes—based on patient-reported health	Median 7.8 y.	Mean age 59 y Only 7% black 41% male United States	No	Donors were not at an increased risk of death or cardiovascular disease.	–
Mjoen.^6^	2014	Donors = 1901 Controls = 32,621	Health Study of Nord-TrØndelag (HUNT) population study.	No—controls were considered fit to donate.	Median 15.1 y.	Mean age 46 y All Caucasian Norway	Yes	Increased risk of all-cause and cardiovascular death	˄
Rizvi.^11^	2016	Donors = 90 Controls = 90	Siblings of donors	Yes—siblings paired.	Mean 5.8 y.	Mean age 37 y 70% male Pakistan	Yes	No difference in rates of ischemic heart disease	–

– No difference, ˄ increase, ˅ decrease.

∗ Symbol indicating results seen in donors.

**Table S2:** Cardiac biomarkers in LKD

Reference	Date	Population	Study Numbers	Control Group	Study Type	Outcome	In Donors∗
**Markers of Inflammation**							
C-Reactive Protein (CRP)							
Kielstein.^65^	2011	LKD	Donors = 24	No controls	Cross-sectional. 1, 6, 12, 24, 72, and 168 h after nephrectomy	Increase postoperatively significantly at 6 h. Peaked at 3 d and then began to decline. Still above baseline at 7 d after nephrectomy.	˄
Huan.^46^	2013	LKD	Donors = 34	No controls	Longitudinal. Baseline and 6 mo	No significant difference between baseline and 6 mo.	–
Kasiske.^38^	2013	LKD and Healthy control	Donors = 201 Controls = 198	Healthy siblings of LKD approached first. Healthy controls meeting LKD criteria.	Prospective. Observational cohort study. Baseline and 6 mo.	No difference between donor and controls	–
Moody.^4^	2015	LKD and healthy controls	Donors = 68 Controls = 56	Healthy controls meeting LKD criteria	Longitudinal. Baseline and 12 mo. Multicenter.	Increased serum high sensitivity CRP in donors compared with controls at 12 mo 1.90 vs. 1.00 mg/dL.	˄
Interleukin-6 (IL-6) and Tumour Necrosis Factor Alpha (TNFα)							
Kielstein.^65^	2011	LKD	Donors = 24	No controls	Cross-sectional. 1, 6, 12, 24, 72, and 168 h after nephrectomy	Increases before CRP. Elevated at 1 h postoperatively then began to decline. Still about baseline at 7 d after nephrectomy.	˄
Huan.^46^	2013	LKD	Donors = 34	No controls	Longitudinal. Baseline and 6 mo	No significant difference in IL-6 or TNFα after donation.	–
**Markers of Myocardial Fibrosis and Left Ventricular Hypertrophy**							
N-Terminal Prohormone of Brain Natriuretic Peptide (NT-proBNP)							
Bellavia.^33^	2015	LKD	Donors = 15 Controls = 15	Italian donors. US age- and gender-matched controls.	Cross-sectional. Measurements at least 5 y after donation.	No difference between donors and controls	–
Moody.^4^	2015	LKD and healthy controls	Donors = 68 Controls = 56	Healthy controls meeting LKD criteria	Prospective, longitudinal. Baseline and 12 mo. Multicenter.	No difference between donors and controls.	–
Altmann.^5^	2017	LKD	Donors = 23	No controls	Prospective, cohort study. Baseline, 4 mo and 12 mo.	No difference after donation.	–
Aldosterone and Angiotensin II							
Bellavia.^33^	2015	LKD	Donors = 15 Controls = 15	Italian donors. US age- and gender-matched controls.	Cross-sectional. Measurements at least 5 y after donation.	No difference in either aldosterone or angiotensin II between donors and controls	–
Moody.^4^	2015	LKD and healthy controls	Donors = 68 Controls = 56	Healthy controls meeting LKD criteria	Prospective, longitudinal. Baseline and 12 mo. Multicenter.	No difference in aldosterone between donors and controls.	–
Renin							
Bellavia.^33^	2015	LKD	Donors = 15 Controls = 15	Italian donors. US age- and gender-matched controls.	Cross-sectional. Measurements at least 5 y after donation.	No difference between donors and controls.	–
Moody.^4^	2015	LKD and healthy controls	Donors = 68 Controls = 56	Healthy controls meeting LKD criteria	Prospective, longitudinal. Baseline and 12 mo. Multicenter.	No difference between donors and controls.	–
High-Sensitivity Troponin							
Moody.^4^	2015	LKD and healthy controls	Donors = 68 Controls = 56	Healthy controls meeting LKD criteria	Prospective, longitudinal. Baseline and 12 mo. Multicenter.	Increase in detectable serum hs-cTnT≥5 ng/L in donors 21% vs. 2%	˄
α-Klotho							
Ponte.^49^	2014	LKD	Donors = 27	No controls	Cross-sectional, observational. 0, 1, 2, 3, 180, and 360 d after donation.	Circulating klotho levels remained lower over a sustained period.	˅
Thorsen.^43^	2016	LKD CKD stage healthy controls	Donors = 35 CKD 3 = 22 CKD 4 = 18 CKD 5 = 20 Controls = 35	Colleagues and friends of the authors.	Cross-sectional, observational, single-center.	No difference between donors and controls. Lower levels seen in patients with advancing CKD.	–
Fibroblast Growth Factor 23 (FGF23)							
Young.^48^	2012	LKD	Donors = 198 Controls = 98	Known to the LKD. Health status based on patient recall.	Cross-sectional. Multi-center.	Serum FGF23 was increased in donors compared with controls (38.1 vs 29.7 pg/mL).	˄
Huan.^46^	2013	LKD	Donors = 34	No controls	Prospective, longitudinal. Baseline and 6 mo.	FGF23 levels increased at 6 mo compared with baseline 54.0 ± 27.9 RU/mL vs. 70.0 ± 32.9 RU/mL.	˄
Ponte.^49^	2014	LKD	Donors = 27	No controls	Cross-sectional, observational. 0, 1, 2, 3, 180, and 360 d after donation.	No change significantly after donation. At 180 d, there was no change in FGF23 levels compared with baseline.	–
Moody.^4^	2015	LKD and healthy controls	Donors = 68 Controls = 56	Healthy controls meeting LKD criteria	Prospective, longitudinal. Baseline and 12 mo. Multicenter.	Increase significantly from 67–84 RU/mL after donation.	˄
Thorsen.^43^	2016	LKD CKD stage healthy controls	Donors = 35 CKD 3 = 22 CKD 4 = 18 CKD 5 = 20 Controls = 35	Colleagues and friends of the authors.	Cross-sectional, observational, single-center.	Nonsignificantly higher in donors compared with controls. Increased as renal function deteriorated.	–
Kasiske.^47^	2016	LKD	Donors = 182 Controls = 173	Matched controls	Prospective, longitudinal. Baseline, 6 mo and 36 mo after donation.	Serum FGF23 levels at 6 and 36 mo were higher than controls.	˄
Amino-Terminal Peptide of Procollagen III (PIIINP) and Procollagen Type I N Terminal Propeptide (PINP)							
Bellavia.^33^	2015	LKD	Donors = 15 Controls = 15	Italian donors. US age- and gender-matched controls.	Cross-sectional. Measurements at least 5 y after donation.	Elevated PIIINP levels seen in donors 5.8 (5.4–7.6) μg/L vs. 1.1 (0.9–1.3)mg/dL.	˄
Kasiske.^47^	2016	LKD	Donors = 182 Controls = 173	Matched controls	Prospective, longitudinal. Baseline, 6 mo and 36 mo after donation.	PINP concentrations were higher at 6 mo than paired normal controls (24.3% and 8.9%). No difference at 36 mo.	˄
Altmann.^5^	2017	LKD	Donors = 23	No controls	Prospective, cohort study. Baseline, 4 mo and 12 mo.	Increase in PIIINP donors seen at 12 mo 0.45 ± 0.11 ng/mL vs. 0.56 ± 0.14 ng/mL	˄

LKD; living kidney donors.

– No difference, ˄ increase, ˄ decrease.

• Klotho is a transmembrane protein associated with FGF23 signaling.

• Procollagen type III N-terminal is involved in fibroblast activation.

• Fibroblast growth factor 23 (FGF23) is a phosphaturic hormone important in phosphate homeostasis, elevated early in CKD, and recently implicated as a cause of left ventricular hypertrophy in CKD in a series of animal and human studies.^1^

∗ Symbol indicating results seen in donors.
